# Grand Challenge in Medical and Surgical Rehabilitation: From Mechanisms to Evidence Based Rehabilitation Programs

**DOI:** 10.3389/fresc.2020.634942

**Published:** 2021-03-12

**Authors:** Christoph Gutenbrunner

**Affiliations:** Department of Rehabilitation Medicine, Hannover Medical School, Hanover, Germany

**Keywords:** medical rehabilitation, surgical rehabilitation, mechanism of rehabilitation intervention, translational research, clinical trials, rehabilitation programs

## Aspects of the Development of Rehabilitation Medicine

Looking back to the roots of modern Rehabilitation Medicine, 3 main streams can be identified.

1. The first one is the need to take care of war victims. This led to a kind of booster rehabilitation service during and after various wars (e.g., *the First and Second World Wars in central Europe or the Vietnam War in The US*) (1). However, past and present natural disasters have also accelerated the implementation of rehabilitation services in affected countries or regions that could be observed; an example would be in Sichuan Province in China after a deleterious earthquake in 2008. In the modern world, this stream can be seen as the root cause of post-acute and long-term rehabilitation after injury and trauma (2).
2. The second stream consists of care programs for children with congenital disabilities, namely deformities in the musculoskeletal system. This type of care dynamically developed in the Nineteenth century (3). Though not many historical studies exist, to some extent, the modern understanding of the provision of rehabilitation services is fundamental from a human rights perspective (4). It can be interpreted as the basis for the modern understanding of rehabilitation as a health strategy to reduce disability as a lived experience of persons with impairments (2, 5).
3. The third stream originates from the tradition of health resort medicine and treatment concepts that use balneological and physical modalities (6). These types of treatment have had a central role in the treatment of chronic health conditions since the Nineteenth century and were then implemented in the modern social and health insurance systems (7). It was developed in central, southern, and eastern Europe; however, some of the traditions were used in the UK and the US. In Europe, it became the basis for the rehabilitation of people with chronic health conditions and to prevent the need for health-related early pension payment. Conceptually, this stream was the basis of the development of rehabilitation concepts for persons with chronic and progressive health conditions (8).

Taking these streams together, today there is strong agreement that rehabilitation must be available during all phases (a*cute, post-acute, and long-term care*) and at all levels (*primary, secondary, and tertiary level*) of health care (8). It has been defined as an essential part of Universal Health Coverage (9) and has been labeled “the health strategy of the twenty-first century” (2). These historical and conceptual reflections on rehabilitation should not lead to underestimation of the importance of health interventions that target the improvement of body functions and structures, activities, and participation. From a scientific perspective, many aspects have to be investigated (10). This includes biomolecular mechanisms of the repair and healing processes, the mechanisms of action of applied physical and chemical modalities, translational research, clinical trials on effects and effectiveness of rehabilitation interventions, and the applicability of rehabilitation programs in healthcare settings. Such research has, apart from its generic aspects, a condition-specific focus, and it is of major importance to a wide range of medical and surgical conditions.

## Main Streams and Gaps in Basic and Clinical Rehabilitation Sciences

Looking at the topics published in scientific journals in the field of rehabilitation medicine, it becomes evident that diseases of the nervous and musculoskeletal systems as well as cardiac and respiratory conditions are predominant areas of research (11). The same profile results, if the abstract submissions to scientific congresses in the rehabilitation field are analyzed (12). Assuming that health-related rehabilitation measures aim at reducing impairments and facilitate functioning independent of the underlying health condition, the focus of rehabilitation medicine must be much broader. A few examples may illustrate this:

- Mental disorders, including depression and anxiety, are frequent health conditions and have a high impact on participation in society and vocational performance (13, 14). Persons with mental disorders have a high need for a wide range of rehabilitation interventions, such as psychotherapy, physical exercise, behavioral treatments, social counseling, and medications. Not much has been published on the outcomes of such multimodal rehabilitation programs, the effects of single interventions (*incl. dosage*), the (*synergistic*) effects of combined treatments, and the duration of rehabilitation interventions. Additionally, knowledge of predictors, age- and gender-specific effects, and the adherence of effects need further elucidation.
- Another field that has not yet sufficiently investigated is rehabilitation for children with specific health conditions (*with the possible exceptions of cerebral palsy and juvenile rheumatoid arthritis*). The gaps are obvious when looking at children with cancer and mental disorders (15, 16). With regard to mechanisms, it should be taken into account that repair mechanisms and the immediate effects of interventions may differ significantly with age and developmental phase. Last but not least the inclusion of parents and other family members into rehabilitation programs must be the focus of research (17).
- Bladder and prostate surgery are other examples of the need for surgical rehabilitation. A number of post-surgical symptoms have a high impact on the patient's quality of life (18). These range from incontinence, external urination systems, sexual dysfunction, fatigue, and mental health problems (19). This demonstrates the need for highly specialized rehabilitation approaches. The effectiveness and efficacy of such interventions must be evaluated. This is also the case for treatment combinations. Another issue from the point of view of a clinician is rehabilitation intervention for sexual functions. Last but not least, research on the impact of surgical techniques on long-term functional outcomes should be investigated more.
- Another recent topic for rehabilitation medicine research is long-term impairment following SARS-CoV-2 infection (*addressed as “post-COVID-19-Syndrome” or “long-term COVID-19”*) (20). Besides respiratory dysfunction, various symptoms have been observed, such as disturbances of smell and taste, dysfunctions in cognition, memory, and motor coordination, muscular pain, fatigue, and depression (21). In principle, all these symptoms are available, but we do not know if this applies to this type of pathology or if any specific strategies must be developed. As it is a new disease, research is needed to elucidate the frequency and severity of symptoms, its impact on participation, and the effectiveness of interventions.

Other conditions as well as rehabilitation of persons with multimorbidity, rehabilitation for old people and patients with different types of cancer could have been taken as examples.

With regard to mechanisms, the knowledge on neuroplasticity and brain repair, training of muscle functions, and influence on tropho-trophic tissues of the musculoskeletal system as well on cardiovascular, respiratory, and metabolic functions and its relevance for rehabilitation approaches have grown dramatically during the last decades; other pathophysiological and repair mechanisms have received much less attention. Examples for this are possible influences on the biomolecular mechanisms of wound healing, mechanisms of pain chronification, and the immune response.

Another issue is that rehabilitation research—similar to the implementation of rehabilitation programs—focuses on the most frequent health conditions, such as back pain, osteoarthritis, diabetes, COPD, myocardial infarction, etc. The development of rehabilitation programs for rare diseases and the research about it is extremely scarce. From an ethical perspective and with regard to scientific relevance, this should be changed (22), and research should be facilitated. This must include an assessment of rehabilitation needs too.

## Future Steps in Medical and Surgical Rehabilitation

The abovementioned examples demonstrate that the field of medical and surgical rehabilitation needs much more attention in research. To conceptualize such fields, more systematic consideration is needed.

For efficient rehabilitation, goal setting has an important role (23). For a systematic goal-setting process that must be based both on the nature and extent of limitations in functioning as well as the patients' perspectives and individual goals, an appropriate assessment is crucial (24). Many assessment tools, including testing of body functions and activities as well as checklists and questionnaires, are available (25, 26). These mostly include condition-specific and functioning-specific aspects. Most condition-specific tools concentrate on frequent rehabilitation diagnoses, such as disorders of the musculoskeletal and nervous system as well as cardiovascular and lung pathologies. There is a need for the development of condition-specific tools or modules for less frequent pathologies. Limb amputation, surgical conditions, and even the post-COVID-19 syndrome are examples.

In many clinical fields, the treatment strategies and intervention emerge very dynamically. This is the case for surgical techniques, pharmacological treatments, and cognitive-behavioral approaches. It is extremely important to investigate how this influences rehabilitation strategies and—on the other hand—how rehabilitation needs may be modified. Some examples for that are the implementation of endo-exo-prothesis after limb amputation, biologicals in the treatment of rheumatoid arthritis, organ transplantation surgery, and others. Furthermore, rehabilitation technology and assistive devices as well as robotics and it-approaches are developing fast. Both aspects must be considered in designing rehabilitation approaches and must be investigated in clinical trials.

Condition-specific rehabilitation programs may distract the attention on so-called non-specific symptoms that are frequently linked to the condition that is seen as an indication for rehabilitation. One example is the phenomenon of fatigue that can be described both as physical and mental fatigue (27). Other such symptoms may be sleep disturbance, widespread pain, thermal discomfort, and a depressed mood. These symptoms or dysfunctions can be observed in patients with diverse pathologies, e.g., cancer (*including post-treatment phase*), infectious diseases, musculoskeletal pain disorders, metabolic disorders, and others. Recently, it has been described that post-COVID-19 syndrome to a high extent also includes suchs symptoms (21). From the point of view of pathomechanisms, responses of the immune system to the primary pathology may play a role (28). On the other hand, most non-specific symptoms are related to autonomous regulation, namely, the hypophysis-pituitary-adrenal axis (29). Last but not least, specific hormonal dysregulation (e.g., *in cancer*) and mediators (e.g., *in chronic widespread pain*) are involved (30–32). As non-specific symptoms may have a major impact on quality of life and hinder participation, rehabilitation must focus on these. Both, studies on mechanisms as well as on programs and outcomes are required. Related to this, studies on biomolecular mechanisms as well as on adaptation and repair mechanisms need to be performed and integrated into a concept of translational research. This also must include mechanisms of treatment effects both as single intervention and as serial applications.

Last but not least the organization of rehabilitation services and their interfaces with other health services may significantly influence service quality and outcomes (33). Here, it is also important to reflect and investigate what specific requirements rehabilitation services must fulfill in order to meet the needs of medical and surgical conditions (e.g., *cancer, mental disorders, organ transplantation, and others*).

All in all, it can be seen that the whole spectrum of rehabilitation research “from cell to society” (10) is of relevance for a scientific approach toward improvements in rehabilitation for medical and surgical conditions. Taking such a broad approach will need careful interpretation and linking of different aspects. It will contribute to better provision of care and outcomes for patients in need of rehabilitation.

## Author Contributions

The author confirms being the sole contributor of this work and has approved it for publication.

## Conflict of Interest

The author declares that the research was conducted in the absence of any commercial or financial relationships that could be construed as a potential conflict of interest.
